# Psychometric properties of the Persian version of the weight-related experiential avoidance (AAQW): overweight and obese treatment seeker at the clinical setting

**DOI:** 10.1186/s12888-021-03352-6

**Published:** 2021-07-05

**Authors:** Mohammad Reza Pirmoradi, Ali Asgharzadeh, Behrooz Birashk, Banafshe Gharaee, Razieh Salehian, Ali Reza Ostadrahimi, Abolfazl Akbarzadeh

**Affiliations:** 1 School of Behavioral Sciences and Mental Health (Tehran Institute of Psychiatry), Iran University of Medical Sciences, Tehran, Iran; 2 Rasoul-e Akram Hospital, Iran University of Medical Sciences, Tehran, Iran; 3 Nutrition Research center, Tabriz University of Medical Sciences, Tabriz, Iran; 4 Tabriz University of Medical Sciences, Tabriz, Iran

**Keywords:** Weight-related experiential avoidance, Psychometric properties, Overweight, Obesity, Persian version

## Abstract

**Background:**

The present study aimed to investigate the psychometric properties of the Persian version of the weight-related experiential avoidance (AAQW) in overweight and obese treatment seeker in the clinical setting.

**Methods:**

This sample consists of 220 male and female overweight or obesity treatment seeker from Overweight and obesity centers who agreed to fill out the self-reported measures.

**Results:**

Confirmatory factor analysis (CFA) supported 3-factor structures of AAQW, including (weight as a barrier to living, Food as Control, and weight-stigma). Furthermore, the internal consistency of AAQW indicates an acceptable range (α = .70); Also, expected associations between AAQW and external correlates (e.g., BES, AAQ-II, KIMS, BDI-II, and CFQ) supported the measure’s convergent validity in a sample of overweight and obese treatment seeker in the clinical setting.

**Conclusions:**

Overall, our study offers that the Persian version of weight-related experiential avoidance has psychometrically valid and reliable tools to assess experiential avoidance. Furthermore, weight-related experiential avoidance is associated with higher severity of binge eating symptoms, higher psychological inflexibility levels, experiential avoidance, and more cognitive fusion and depression symptomology.

## Background

In recent years the psychological treatment and conceptualization of obesity and overweight have been increased. Several weight-related psychological factors (such as experiential avoidance, emotional eating, impulsivity, and strict eating control) may reflect weight-related experiential avoidance patterns, which lead to low quality of life in overweight and obese individuals [1].

Experimental avoidance is associated with a reluctance to contact unpleasant internal experiences related to weight and eating (such as a craving for food, fatigue, self-labeling, or weight-based stigma) and an attempt to avoid, control, or change them [2]. In the conceptualization of overweight and obesity based on Acceptance and Commitment Therapy, experiential avoidance is considered a primary factor of problematic behaviors. Overweight and obese individuals feel more negative emotions by using avoidance strategies such as more diet and food avoidance. Therefore, they learn to eat as a coping mechanism for short-term avoidance of painful emotions. Because emotion regulation strategies and psychological outcomes are mediated by experiential avoidance, negative emotions are associated with increased experiential avoidance, which increases behaviors related to overeating and self-avoidance [3].

As the ACT model’s objective is to increase psychological flexibility and decrease experiential avoidance, it is essential to develop instruments that assess the changes in these processes [4]. To measure psychological flexibility, Hayes et al. (2004) developed an Action and Acceptance Questionnaire (AAQ) that were used in widely different research [5]. However, the researchers modified versions of AAQ in the areas of particular when such a tool is used to measure change processes [6]. As a result, AAQ-II is the most used instrument to measure psychological flexibility [7]. However, this technique is a general measurement and evaluates mainly anxiety and depression, which raises some questions when applied to processes of changes in specific problems.

Lillis [2007] found that a weight-related version of the AAQ seems needed to assess therapeutic change and outcomes in weight loss programs and weight maintenance. In this sense, Lillis & Hayes [2008] developed the more specific Acceptance and Action Questionnaire for Weight-Related Difficulties (AAQW) that specifically assess the avoidance and inflexibility of thoughts and feelings about weight in overweight and obese individuals [6].

The original version of AAQ-W-22 showed good psychometric qualities and good test-retest reliability and had a unifactorial structure [6]. Therefore, other studies were needed to study factor structure. Further psychometric properties of AAQ Weineland et al. [2013] examined the psychometric properties (test-retest, validation, internal consistency, and factor structure) of the AAQW in a bariatric surgery population in Sweden. The Swedish version of AAQW-20 had a five-factor structure (food as control, body acceptance, self-stigma, self-efficacy, and emotional avoidance), and they showed that the AAQ-W appears to be a psychometrically sound measure that useful for researchers and clinicians. However, They suggested AAQ-W needs more research [8].

The latest research about the psychometric properties of the AAQW is Palmeira et al. [2016] studies of the Portuguese version of AAQW [1]. The results of the factor analysis Portuguese version of AAQW did not support the unifactorial structure of the original version [6] and five factors found in the Swedish version of AAQW [1]. Thus, the Portuguese version of AAQW was constituted by 10 items distributed by three factors (food as control, emotional avoidance, and self-stigma. Portuguese version of AAQW had good internal consistency (α = .81) and good convergent and divergent validity [1].

In the present study, due to the growing trend of Acceptance and Commitment Therapy (ACT) application for treating overweight and obesity in Iran and lack of appropriate studies, we aimed to evaluate the three factors factor structure, internal consistency, and criterion validity of the Persian version of the weight-related experiential avoidance (AAQW) in overweight and obese treatment seeker in the clinical setting. We hypothesize that the Persian version of AAQW as psychometrically valid and reliable tools could be a helpful tool for clinicians to measure experiential avoidance’s outcome and treatment process.

## Methods

### Participants

This sample consists of 220 males and females with overweight or obesity treatment seekers from overweight and obesity centers affiliated to Tabriz University of Medical Sciences. The inclusion criteria included the participants’ willingness to participate in the investigation. Also, all males and females with overweight or obesity treatment seekers over the age of 18 were eligible to participate. When the participants refer to the overweight and obesity centers, the assessments are performed. According to Meyers, Gamst, and Guarino [9], to perform the CFA for each item in the scale, 5–10 samples are adequate. Concerning the number of AAQW items (22 items), *N* ≥ 220 was sufficient [9]. (Response rate of more than 95%). Missing Values within the data file were replaced with the mean series method Histograms and boxplots method were also used to detect outliers. Data of 20 participants were deleted because of outliers and lack of incomplete data.

### Measures

Acceptance and Action Questionnaire for Weight-Related Difficulties (AAQW) the questionnaire consists of 22 Likert items with seven response options: 1 (never true), 2 (very seldom true), 3 (rarely true), 4 (sometimes true), 5 (frequently true), 6 (almost always true), and 7 (always true) developed by Lilis and Hayes [2007] to assess psychological inflexibility. The original version of AAQ-W showed good internal consistency (α = 0.86) [6].

Cognitive Fusion Questionnaire (CFQ) This questionnaire was developed by Gillanders et al. [2014] and had 7 questions and is graded like “never true (1) to be absolutely true (7)” and higher scores reflect more cognitive integration. CFQ showed good initial evidence of factor structure, reliability, validity, and sensitivity to change in different samples of 1800 people. In addition, the reliability of the Persian version of the Cognitive Fusion Questionnaire (CFQ) was reported good range (α = 0.86) [10].

The Binge Eating Scale (BES) The BES is a self-administered questionnaire composed of 16 items: 8 items that describe behavioral manifestations (for example, eating fast or consuming large amounts of food) and eight items on associated feelings and cognitions (for example, fear of not stopping eating). Each item has a response range from 0 to 3 points (0 = no severity of the BES symptoms, 3 = serious problems on the BES symptoms [11]. The Persian version of the Binge Eating Scale (BES) showed good internal consistency (α = 0.71 and 0.85, respectively) [12].

Beck’s Depression Inventory- Second Edition (BDI-II) The BDI-II is one of the most widely used self-report instruments for assessing the symptoms of depression. The Persian version of BDI-II showed a high internal consistency (α = 0.87) [13, 14].

Acceptance and Action Questionnaire (AAQ-II) was developed by Bond et al. [2011] and had 10 questions that measure acceptance, empirical avoidance, and psychological inflexibility. Higher scores indicate greater psychological flexibility [7]. The internal consistency of the Persian version of the Acceptance and Action Questionnaire (AAQ-II) was reported in a good range (α = 0.89), [15].

Kentucky Inventory of Mindfulness Skills (KIMS) is a 39 self-report 5 point Likert item ranging from 1 (never or very rarely true) to 5 (almost always or always true). Items reflect the mindfulness skills or the absence of that skill. Thus, high scores reflect more mindfulness. The results of the psychometric analysis on the participants showed that this questionnaire has a high internal consistency (α = 0.73), and Cronbach’s alpha coefficients of the subscales of observation, descriptiveness, concentration, and acceptance were .91, .84, .83, and .87, respectively [16]. The Persian version of the Kentucky Inventory of Mindfulness Skills (KIMS) indicated the six dimensions and has a satisfactory internal consistency (α = .81 to α = .93) [17].

### Procedures

In the first step, the English version (source language) of the AAQW was received by Email from the primary constructor, then forward translation (English into Persian) done by two independent bilingual translators. In stage 2 then translators and researchers synthesized the translations. In the third step, the back translation from Persian to English was conducted by the blinded translator into the original version. In the next phase, the expert’s committee assesses equivalence between the original and target versions. 30 pilot participants filled out the AAQW and examined the clarity and simplicity of all of the exact and apprehensible items for pilot participants. After the pilot phase, the AAQW was filled out by the overweight or obesity treatment seekers. The Iran University of Medical Sciences ethics committee confirmed the study that the approval code is IR.IUMS.REC 1395.95–03–121-29,331. After the explanation of study purposes and assurance of confidentiality, participants provided. All analysis was carried out by IBM SPSS v. 18.0 [18] and Mplus 5.1.

### Data analysis

In the current research study, we used the frequency table and box plots to identify outlier data. Given that the missing data accounted for less than 5% of the total data, replacing missing values ​​with the mean method was used to replace the missed data. To test the normality of the data, skewness and kurtosis indices was assessed. The questions’ skewness ranged from − 0.898 to 1.469, and their kurtosis went from − 1.479 to 1.204. These values ​​are acceptable (SK < | 3 | and Ku < | 8–10 |). To test the absence of multivariate outliers, the Mahalanobis distance index for each individual was calculated. Although there were many multivariate outliers based on this index; however, based on the recommendation that preserving outdated data makes the data representative, these data were not deleted [19, 20]. Finally, the correlation matrix between the questions examined the multicollinearity assumption. The correlation between the questions ranged from 0.126 to 0.472. This assumption is fulfilled if the correlation between the questions does not exceed 0.85 [21].

Next, CFA was conducted through Mplus Version 5.1. First, to evaluate the factors of the AAQW. Then, Cronbach’s alpha coefficient was used to assess the internal consistency of the AAQW. Finally, convergent and divergent validation, Pearson correlation coefficients were examined between its scores and alternative measure (e.g., The Binge Eating Scale, the Acceptance and Action Questionnaire-II, the Kentucky Inventory of Mindfulness Skills, Beck Depression Inventory-II, Cognitive Fusion Questionnaire).

## Results

137 (68.5%) of participants were female, and 54 (27%) were male. Also, 9 (4.5%) of them did not specify their gender. 44 (22%) were single, 150 (75%) were married, and 6 (3%) no identified marital status. 12 people (6%) have 1–5 grade literacy level, 56 people (28%) have 6–13 grade literacy level, 90 people (45%) have bachelor’s degree, 25 people (12.5%) have master’s degree and 13 people (6.5%) had a doctorate. 4 people did not declare their level of education. The mean age of participants was 39.05 years (SD = 10.75), their mean height was 164.10 cm (SD = 13.82), and their mean weight was 74.50 kg 13.82 (SD = 15.01). Also, the mean BMI index of the participants was 27.70 (SD = 5.18). (*Χ*^2^ [4] = 31.92, *p* < .001) The distribution of the studentized smallest chi-square plays an important role in testing for the homogeneity of variances and the ranking and selection procedures [22].

### Confirmatory factor analyses

In this study, the three-factor model was evaluated using second-order confirmatory factor analysis. Fit indicators for this model are reported in Table 1. As can be seen, many fit indicators such as IFI, TLI, and CFI are not in the proper fit range for this model. In the hypothetical three-factor model, all factor loads except the factor load of item 9 were significant. However, item 9 on the factor (weight as a barrier to living) with a value of λ = − .07 had no significance (*P* = .435). Therefore, this question was removed, and the second time confirmatory factor analysis was performed on the questions. The results of the three-factor model fit indices are reported by omitting item 9 in Table 1. As can be seen, all fit indicators have improved. For this model, all indicators except TLI are within the appropriate fit of the model. The value obtained for TLI is also very close to the acceptable range (TLI ≥ .90). Therefore, in general, the appropriateness of the final factor structure can be confirmed.

**Table 1:** Fit indices of confirmatory factor analysis

Models	chi-square	P	CMIN/DF	RMSEA	GFI	IFI	TLI	CFI
three-factor model	70.95	.001	2.15	.076	.93	.87	.82	.86
three-factor model by omitting item 9 (AAQW-9)	46.13	.006	1.84	.065	.95	.92	.88	.92

Note. CMIN/DF: Relative chi-square relative chi-square; RMSEA = Root Mean Error of Approximation; GFI = Goodness of Fit Index; IFI = Incremental Fit Index TLI = Tucker-Lewis Index; CFI = Comparative Fit Index

**Fig. 1:**
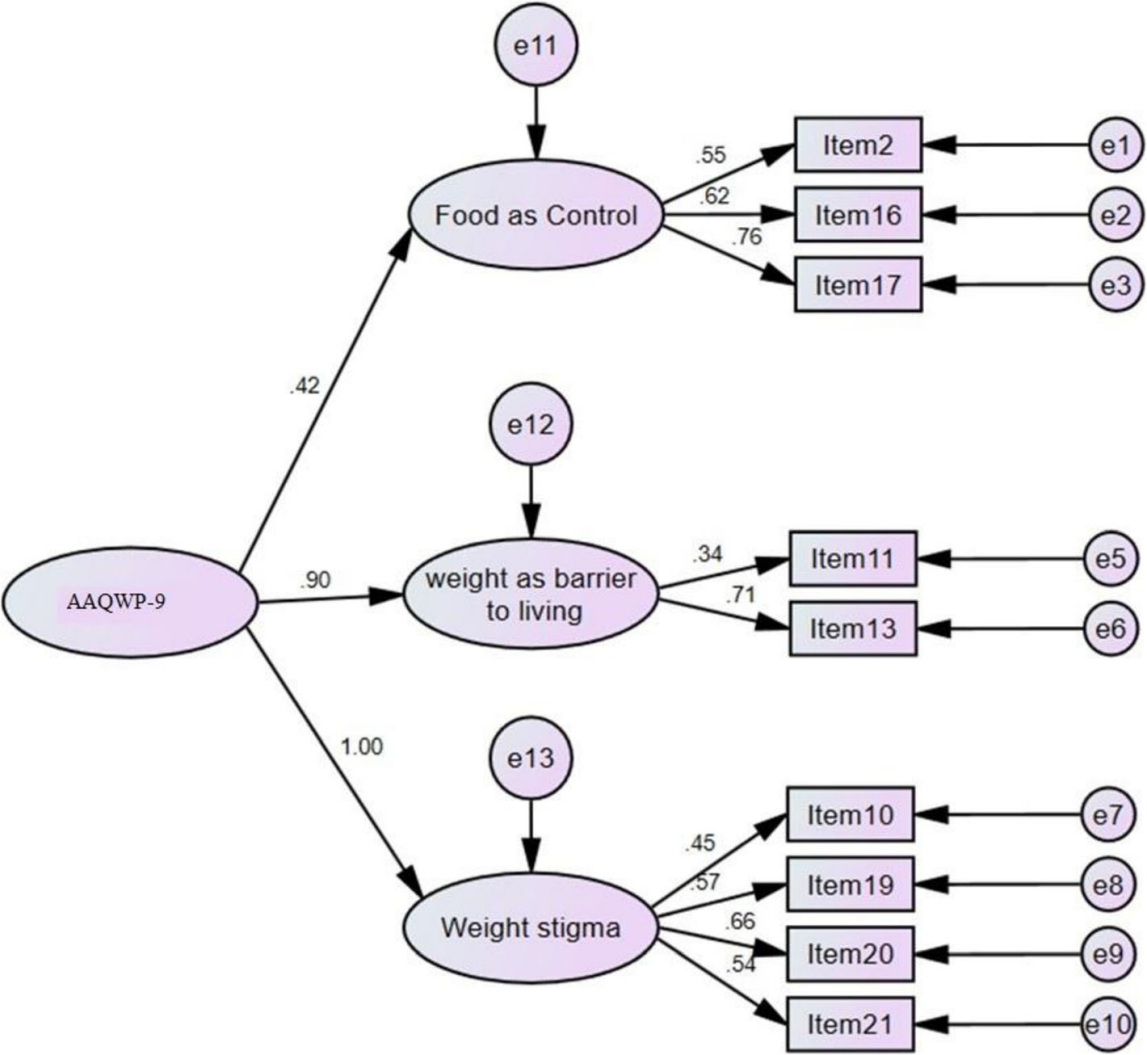
The final factor structure of AAQW-9. Item 2 = negative feelings, Item 16 = My eating urges control me, Item 17 = I need to get rid of my eating urges to eat better, Item 11 = If I’m overweight, I can’t live the life I want to, Item 13 = If I gain weight, that means I have failed, Item 10 = Other people make it hard for me to accept myself, Item 19 = If I eat something bad, the whole day is a waste, Item 20 = I should be ashamed of my body, Item 21 = I need to avoid social situations where people might judge me

The factor structure related to the final factor analysis is shown in Fig. 1. Again, all values are significant at the level of *P* < 0.01.

### Reliability

Cronbach’s alpha was used to evaluate the reliability of the AAQW-9 using internal consistency (cut-off of .70 is considered suitable) [23]. Therefore, the Cronbach’s alpha value for the total AAQW-9 by removing item 9 was (α = .70), which indicates the acceptable reliability of AAQWP-9.

### Convergent and divergent validation

Pearson correlation was conducted to evaluate the convergent and divergent validity of AAQW-9 and its three subscales with The Binge Eating Scale (BES), The Acceptance and Action Questionnaire-II (AAQ-II), Kentucky Inventory of Mindfulness Skills (KIMS) Beck Depression Inventory-II (BDI-II), and Cognitive Fusion Questionnaire (CFQ), the results are reported in Table 2.

**Table 2:** AAQWP-9 subscale Pearson correlation

	N	BES	AAQ-II	KIMS	BDI	CFQ
AAQW-9-total	200	.483^**^	.340^**^	−.246^**^	.313^**^	.326^**^
Food as control	200	.376^**^	.138	−.075	.167^*^	.139
Weight stigma	200	.415^**^	.324^**^	−.297^**^	.295^**^	.315^**^
Weight as a barrier to living	200	.253^**^	.283^**^	−.133	.215^**^	.254^**^

*Note.* ** *P* < 0.01 * *P* < 0.05 AAQWP-9 = the Persian version of the weight related experiential avoidance; BES = The Binge Eating Scale; AAQ-II = the Acceptance and Action Questionnaire-II; KIMS = Kentucky Inventory of Mindfulness Skills; BDI = Beck Depression Inventory-II; CFQ = Cognitive Fusion Questionnaire

## Discussion

The main objective of this study was to investigate the three-factor structure, internal consistency, and criterion validity of the Persian version of the weight-related experiential avoidance (AAQW) in overweight and obese treatment seeker in the clinical setting, which assesses experiential avoidance and psychological inflexibility related to difficult thoughts and feelings about weight [6]. Our results confirmed the three-factor structure of AAQW with 9 items. Furthermore, internal consistency and criterion validity supported that the Persian version of the weight-related experiential avoidance (AAQW) validity and reliability in a sample of overweight and obese treatment seeker individuals.

The literature has consistently demonstrated that experiential avoidance and psychological inflexibility are closely related to various psychological disorders and health problems. Specifically, concerning obesity, there are still few studies that evaluate the role of these psychological processes. However, the current research points to the fact that these processes are associated with worse quality of life, disturbed eating behavior, and higher psychopathology levels.

Our research is based on three previous English, Swedish, and Portuguese languages [1, 3, 6]. The results of the factor analysis did not allow replicating the last structure factorial found in the original version of the instrument, which contained unifactorial 22 items [6], five factors 22 items [3] the latest three factors 10 items [1]. Nevertheless, the Persian version of AAQW revealed a factorial structure composed of three factors 9 items, which correspond: food as a control (items: 2, 16, 17); weight as barriers to living (items: 11, 13); weight stigma (items: 10, 19, 20, 21). The result of the present study’s factor structure in line with Palmeira, Cunha, et al., [2016] studies [1]. But the Persian version of the weight-related experiential avoidance (AAQW) led to 9 items, which we called the Persian version of the weight-related experiential avoidance (AAQW-9). It is noteworthy that we considered RMSEA scores below .05 to indicate a good fit and scores between .05 and .08 indicating acceptable fit. A TLI and CFI score of .95 or above indicates excellent fit, and scores of .90 or more indicate a good fit (Bentler, 1990; Hu & Bentler, 1999). Concerning χ2, a good fit is shown when χ2 (*p* > .05) and χ2/df ≤ 2, whereas χ2/df ≤ 3 is indicative of an acceptable fit (Schermelleh-Engel et al., 2003).

The AAQW-9 total had acceptable internal consistency, while the AAQW-9 subscales (weight as a barrier to living, Food as Control, and weight-stigma) presented a questionable to unacceptable internal this is probably due to the small number of items in both scales. Furthermore, these results are similar to those found in the previous version [1, 6, 8]. The analysis of items 10, 11, 19 and showed low correlation values, and it seems that Persian culture and language do not measure weight as a barrier to living and weight-stigma, and maybe it’s better to replace them with more appropriate ones.

The convergent and divergent validity of AAQW-9 was also explored, and the respective subscales. Consistently with what was found in the literature, the total AAQW-9 was associated with higher severity of binge eating symptoms, higher levels of psychological inflexibility and experiential avoidance, and more cognitive fusion and depression symptomology [1, 6, 8, 24–26]. In this sense, the results suggest that the participants who present more psychological inflexibility related to weight tend to perceive a reduced psychological flexibility pattern and have higher binge eating symptoms, psychological inflexibility, experiential avoidance, more cognitive fusion, and depression symptomology. Therefore, therapeutic interventions that can target these components can reduce obesity and overweight in individuals. In the area of eating behavior, it was verified, as it was expected, that psychological weight-related inflexibility is associated with dietary psychopathology and compulsive eating behaviors, which corroborates the results of previous investigations [1, 6, 8] These results suggest that participants who have more dietary psychopathology, more compulsive eating behaviors, tend to resort more to experiential avoidance to deal with negative internal experiences related to their weight. Indeed, it has been suggested that eating behavior problems can be considered as problems of psychological inflexibility; this experience assesses by food as a controlling factor that Representative negative feeling, eating urges, need to get rid of eating urges to eat better [27].

Regarding the Food factors such as Control and Self-stigma, these showed associations similar to those of the total scale. Of note is the result between the subscale Food as a control and the constraint variable, among which there is a negative and significant association. This means that individuals who report a greater tendency to limit the amount of food they eat to influence their weight or body shape tend to report a lesser tendency to lose control of their diet. Effectively, the restriction has been associated with weight loss and better treatment results, while the tendency towards uncontrolled eating is consistently associated with worse outcomes and problems with eating behavior [28].

Regarding the emotional avoidance subscale that assesses the level at which the person avoids thinking or feeling something unpleasant regarding his weight, this was associated with more disturbed eating behaviors, worse quality of life, and internalized stigma about weight. This result reveals that the more the participants tend to avoid negative internal experiences related to their weight, the more they tend to nurture dysfunctional eating behaviors, report a worse quality of life, and devalue themselves based on their weight. The experiential avoidance assessed by this subscale is associated with these variables negatively, which corroborates the literature, as it demonstrates that this is a psychological factor that negatively influences the health and well-being of the subjects [29].

The weight stigma factor has a much stronger association with binge eating, acceptance, action, mindfulness skills, and depression cognitive fusion variables, which suggests that the level of internalized stigma is more related to the perception of happiness. This result may mean that the more a person devalues ​​himself concerning his weight, the more he tends to consider having a worse quality of life and is affected by weight and the unhappiness he feels about his life.

About the limitations of the present study, it can be pointed out that it is a cross-sectional study, limiting the generalization of the data and does not allow causal relationships. Furthermore, the fact that data collection is only through self-report questionnaires can also be considered another limitation of the study, which may bias some data due to social desirability. In future investigations, it would be pertinent to test the test-retest validity and the discriminative validity of the AAQWP-9. Likewise, it would be relevant to carry out a confirmatory factor analysis, trying to replicate this factor structure, to understand whether it remains unchanged in different general population samples.

## Conclusion

In conclusion, the Persian version of AAQW-9 presents satisfactory psychometric properties to assess weight-related experiential avoidance in overweight and obese individuals. The results also suggest that weight-related experiential avoidance is associated with higher severity of binge eating symptoms, higher levels of psychological inflexibility and experiential avoidance, and more cognitive fusion and depression symptomology.

## Data Availability

The datasets used during the current study are available from the corresponding author on reasonable request.

## Abbreviations

AAQW: the weight related experiential avoidance
CFA: Confirmatory factor analysis
DSM-5: Diagnostic and Statistical Manual of Mental Disorders-5th Edition
α: alpha
CMIN/DF: Relative chi-square relative chi-square
RMSEA: Root Mean Error of Approximation
GFI: Goodness of Fit Index
IFI: Incremental Fit Index
TLI: Tucker-Lewis Index
CFI: Comparative Fit Index
M: Mean
SD: standard Deviation
